# High Fundamental Frequency (HFF) Monolithic Quartz Crystal Microbalance with Dissipation Array for the Simultaneous Detection of Pesticides and Antibiotics in Complex Food

**DOI:** 10.3390/bios12060433

**Published:** 2022-06-20

**Authors:** María Calero, Román Fernández, María García, Marisol Juan-Borrás, Isabel Escriche, Antonio Arnau, Ángel Montoya, Yolanda Jiménez

**Affiliations:** 1 Centro de Investigación e Innovación en Bioingeniería (Ci2B), Universitat Politècnica de València, Camino de Vera s/n, 46022 Valencia, Spain; roferdia@eln.upv.es (R.F.); aarnau@eln.upv.es (A.A.); amontoya@eln.upv.es (Á.M.); yojiji@eln.upv.es (Y.J.); 2 Advanced Wave Sensors S.L., Calle Algepser 24, 46988 Paterna, Valencia, Spain; mgarcia@awsensors.com; 3 Instituto de Ingeniería de Alimentos para el Desarrollo (IIAD), Universitat Politècnica de València, Camino de Vera s/n, 46022 Valencia, Spain; majuabor@iad.upv.es (M.J.-B.); iescrich@tal.upv.es (I.E.)

**Keywords:** immunosensor, HFF-QCMD array, honey, antibiotic, pesticide

## Abstract

As in the case of the food industry in general, there is a global concern about safety and quality in complex food matrices, such as honey, which is driving the demand for fast, sensitive and affordable analytical techniques across the honey-packaging industry. Although excellent techniques such as liquid chromatography-tandem mass spectrometry (LC-MS/MS) are available, these are located in centralized laboratories and are still lacking in speed, simplicity and cost-effectiveness. Here, a new approach is presented where a competitive immunoassay is combined with a novel High Fundamental Frequency Quartz Crystal Microbalance with Dissipation (HFF-QCMD) array biosensor for the simultaneous detection of antibiotics and pesticides in honey. Concretely, thiabendazole and sulfathiazole residues were monitored in spiked honey samples. Results revealed that HFF-QCMD arrays provide a complementary and reliable tool to LC-MS/MS for the analysis of contaminants in these kinds of complex matrices, while avoiding elaborate sample pre-treatment. The good sensitivity achieved (I_50_ values in the 70–720 µg/kg range) and the short analysis time (60 min for 24 individual assays), together with the ability for multiple analyte detection (24 sensor array) and its cost-effectiveness, pave the way for the implementation of a fast on-line, in situ routine control of potentially hazardous chemical residues in honey.

## 1. Introduction

The analysis of contaminants in complex food matrices by current analytical techniques is a difficult task requiring elaborate sample pre-treatment. Honey is an example of a complex food that has attracted great interest in recent years. Because of primary agricultural and livestock activities, bees can be exposed to potentially hazardous chemical residues, contaminating the beehive products and reaching the end consumer. This public health problem is a concern to the authorities, and especially to the beekeeping sector and the scientific community [1,2,3]. The origin of those chemicals comes from veterinary treatments with acaricides, sulfa drugs, antibiotics, etc., necessary to deal with diseases and parasites in bees [4,5]; and from agricultural treatments with pesticides, mainly neonicotinoids [6,7,8,9]. A report by the EFSA (European Food Safety Agency) has confirmed this reality, highlighting the impact of these residues on the health of bees [9], and on that of honey and pollen consumers [10]. Therefore, controlling the presence of chemicals is essential in the marketing of honey. Current regulations are increasingly restrictive, limiting or even prohibiting the presence of these chemicals [11]. The ‘gold standard’ technique for quantifying these residues is the liquid chromatography-tandem mass spectrometry (LC-MS/MS) methodology [12,13,14]. LC-MS/MS provides the lowest limits of detection (LOD) both for pesticides [12] and antibiotics [15]. However, chromatographic techniques are expensive, time-consuming and require highly qualified personnel. These techniques do not routinely allow for the simultaneous analysis of several residues with different chemical properties (i.e., pesticides and antibiotics) in a single assay. LC-MS/MS analyses require an initial extraction step to separate the target compounds from the honey to avoid possible matrix effects that could interfere in the detection process [16]. Since the residue concentrations present at the sample are relatively low, the extraction step is usually tapped for analyte pre-concentration. Because of the dissimilar physicochemical properties of each family of residues of interest, pre-treatment of the sample and even the chromatographic setup must be configured differently when a high resolution is demanded [15,16].

Immunoassays such as ELISA (enzyme-linked immunosorbent assay) [17,18,19,20,21], CLIA (Chemiluminescence Immunoassay) [22] or even RIA (Radioimmunoassay) [23] are ancillary methods frequently used as screening tests. They are based on antibody-antigen recognition, which offers high specificity and sensitivity, as well as cost-effectiveness. However, general immunoassay technologies often require long incubation periods and repeated washing steps that make their automation difficult for on-line sample analysis. Moreover, these analytical methods are only available in centralized laboratories and, consequently, they are not adequate as on-line control tools implanted in situ in a honey packaging industry, which needs automated, simpler, cheaper and faster screening methods that provide LODs as close as possible to those required.

In this scenario, biosensors emerge as complementary and/or alternative methods to the classical ones. Among the different biosensing techniques [24,25], biosensors based on acoustic wave sensors, and particularly Quartz Crystal Microbalance with Dissipation (QCMD), stand out as a real-time and label-free detection tool suitable for the analysis of contaminants in complex matrices, such as honey. QCMD relies on electrically measuring resonance properties (frequency Δ*f* and dissipation Δ*D*) of a quartz crystal resonator [26]. During recent years, clear improvements in LOD and sensitivity have been achieved in High Fundamental Frequency QCMD (HFF-QCMD) sensors [27,28]. The HFF-QCMD principle of operation relies on the reduction in the quartz plate thickness of a classical QCMD [29], resulting in a sensitivity increase and a surface reduction [30]. Recently, individual HFF-QCMD sensors have been combined with a competitive immunoassay and used for pesticide (DDT [31] and carbaryl [32]) and antibiotic (sulfathiazole [33]) detection in honey samples with satisfactory results. However, the use of individual sensors is not feasible for the routine control of multiple simultaneous chemical residues in complex samples. Thanks to their small footprint, it is possible to integrate dozens of HFF-QCMD sensors within the same substrate through the design of monolithic HFF-QCMD arrays [34]. Miniaturized and parallelized elements in the array lead to relevant benefits including high throughput, lower cost per sensor unit, less sample/reagent consumption and faster sensing response [35,36,37].

In this work, a preliminary validation of the HFF-QCMD array as a potential technology for the development of a high throughput screening (HTS) system for multiple analyte detection in complex food samples is shown for the first time. A competitive immunosensor for the simultaneous detection of two compounds belonging to very different chemical families: the fungicide thiabendazole (TBZ) and the antibiotic sulfathiazole (SFZ) in honey samples is presented. Maximum Residue Limit (MRL) established by European Union regulation for TBZ in honey is 50 µg/kg (EC Regulation 2017/1164). In the case of antibiotic residues as SFZ, EU legislation demands complete absence (EC Regulation 37/2010). The analytical performance of the proposed method in terms of LOD, Limit of Quantification (LOQ), accuracy and precision, was compared to LC-MS/MS as reference technique.

## 2. Materials and Methods

### 2.1. Honey Samples, Chemicals and Immunoreagents

Thiabendazole- and sulfathiazole-free honey (supplied by “Beemiel”, Valencia, Spain and checked by chromatographic analysis) was used as “blank honey”. Honey samples were spiked with TBZ (Riedel-de Häen, Seelze, Germany) or SFZ (Sigma Aldrich, Steinheim, Germany) when required.

The reagents used for covalent immobilization of the sensor array were: thiol compounds 11-mercapto-1-undecanol 97% (MUOH) and 16-mercaptohexadecanoic acid 90% (MHDA), 1-ethyl-3-(-3-dimethyl-amino-propyl) carbodiimide hydrochloride (EDC), n-hydroxysuccinimide (NHS) (all of them provided by Sigma-Aldrich Chemie, Steinheim, Germany), and ethalonamine blocking agent (from Sigma, St. Louis, MO, USA). Bovine Serum albumin (BSA) fraction V (Sigma-Aldrich Chemie, Steinheim, Germany) was used to prevent non-specific antibody adsorption to the functionalized surface.

The immunoreagents for SFZ assay (provided by Custom Antibody Service, U2-ICTS-NANBIOSIS; Nb4D group-IQAC-CSIS/CIBER-BBN; Barcelona, Spain) were the following: SA2-BSA AE1 B28 protein-hapten conjugate and 6C11 (batch 8678) purified monoclonal antibody (MAb) against sulfathiazole. The immunoreagents for TBZ assay, BSA–TN3C protein–hapten conjugate and LIB–TN3C13 MAb were previously prepared as described [38].

Nanopure water and pure ethanol were purchased from Panreac Química SLU (Barcelona, Spain). Phosphate buffered saline (PBS) tablets for preparing 0.01 M phosphate buffer containing 0.0027 M potassium chloride and 0.137 M sodium chloride, pH 7.4, at 25 °C were from Sigma Aldrich Química, S.L.U. (Madrid, Spain) and was used as mobile phase in experiments. For cleaning the arrays and some pieces of the microfluidic system we used: a 20% solution of sodium dodecyl sulfate (SDS) (from Fisher Scientific S.L., Madrid, Spain), COBAS Cleaner (provided by Sanilabo S.L., Valencia, Spain), and piranha solution obtained by a mixture of hydrogen peroxide (H_2_O_2_, 50% purity) and sulphuric acid (H_2_SO_4_, 95%) in a 1:3 (*v*/*v*) ratio (both from Merck Life Science S.L.U., Madrid, Spain).

For the regeneration of the HFF-QCMD array we used sodium hydroxide prepared from pellets (98% NaOH) from Sigma Aldrich Chemie (Steinheim, Germany) and 1 M hydrochloric acid from Acros Organics purchased from Fisher Scientific S.L. (Madrid, Spain).

HPLC information about both protocols and regents used are detailed in the Supplementary Materials (Section S1).

### 2.2. HFF-QCMD Array Methodology

#### 2.2.1. HFF-QCMD Array Immunosensor Setup

Arrays of 24 HFF-QCMD sensors were supplied by AWSensors (AWSensors S.L., Valencia, Spain). Arrays are based on a 50 MHz one-sided inverted MESA geometry and were optimized in terms of size, electrode geometry and inverted MESA region thickness for spurious mode suppression and operation in liquids [30,34]. Other constraints imposed by the manufacturing and integration with fluidics and electronics [39] were considered as well.

**Figure 1:**
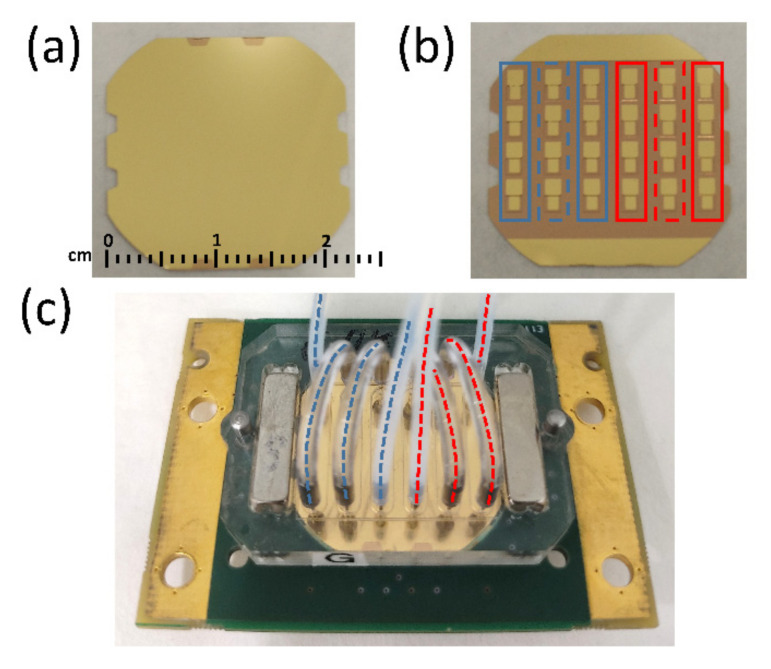
(**a**) Top and (**b**) bottom surfaces of the 24 HFF-QCMD array. (**c**) Array mounted in the flow cell. Blue and red dashed lines in (**c**) indicate the three columns of regions A and P, respectively, that share the flow.

Figure 1a,b show top and bottom surfaces of the array device, respectively. Figure 1c shows the array mounted in the flow measurement cell (Jobst Technologies, Freiburg, Germany). The measurement cell is divided into 6 independent flow channels covering 4 sensors each. Each channel has an inlet and an outlet that can be connected to flow tubing through steel cannulas. Flow connections of the array sensor measurement cell were configured to create two independent flow regions A and P (with 3 columns each) represented in Figure 1c with blue and red dashed lines, respectively.

Both the arrays and the cartridge were cleaned, before surface functionalization, following the previously described protocol [39].

AWS X24 platform (AWSensors) was used for the simultaneous characterization of the resonance frequency *f* and energy dissipation *D* of the 24 elements of the array in real time [40] and the AWS F20 platform (AWSensors) to generate a uniform flow through the array surface. Temperature was controlled and kept at 25 °C and a degasser was used to prevent bubbles.

#### 2.2.2. Detection Format and Array Sensor Functionalization

The indirect competitive immunoassays developed to determine TBZ and SFZ were binding inhibition tests based on the conjugate-coated format described elsewhere [27]. A purposed designed immobilization cell (provided by AWSensors) was used to expose only the first and last columns in the blue and red regions (solid line rectangles in Figure 1b) to surface functionalization reagents for SFZ and TBZ detection, respectively. Each sensor in the central column of both regions was used as the reference sensor of the two neighbors (dashed line rectangles in Figure 1b).

Immobilization protocols were based on those previously reported in references [27,32,33], with the following volumes and concentrations adapted to the array immobilization cell: (a) 100 µL of a 2.5 mM solution of compounds MUOH and MHDA in ethanol (50:1 M ratio); (b) 100 µL of an ethanolic solution of EDC/NHS was incubated for 3.5 h.

#### 2.2.3. Immunoassay Protocol

**Figure 2:**
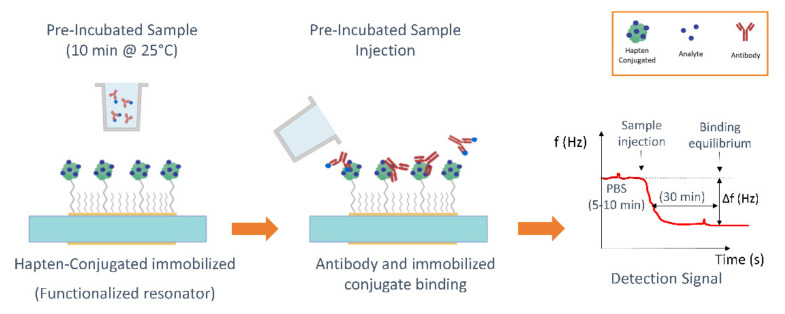
Schematic of the immunoassay protocol.

The inhibition assay protocols were based on those previously reported [27,33]. Briefly, in the first step, a mixture of a fixed concentration of the corresponding MAb with standard solutions of the analyte (2.86 × 10^5^ to 2.86 × 10^−1^ μg/kg) or with the spiked honey samples was preincubated for 10 min at 25 °C. A 20 μL/min continuous flow rate of working buffer (PBS) was pumped through sensors. When a nearly constant baseline was reached (5–10 min), 250 µL of the preincubated mixture were injected over the functionalized immunosensors surface. As the binding between the free antibody and the immobilized conjugate took place, the shifts in *f* and *D* were monitored in real time. Binding equilibrium was reached after ~30 min. The regeneration of the reactive surface to break the active antibody-hapten conjugate binding was carried out by pumping 0.1 M HCl for SFZ and 0.1 M NaOH for TBZ at a flow rate of 125 µL/min. All diluted standards were tested at least three times. Injections corresponding to the maximum signal (absence of analyte in the dilution) were run every two standard solution injections for signal normalization and for evaluating the functionalization quality. See in Figure 2 a schematic of the protocol.

## 3. Results and Discussion

### 3.1. Immunoassay Optimization: Selection of the Immunoreagent Concentrations

The optimal combination of hapten-conjugate and monoclonal antibody concentrations was investigated to find an optimal trade-off between a good signal-to-noise ratio for the lowest analyte concentration (resonance frequency shift signal of ~1000 Hz), and a minimum immunoreagent consumption [32,33]. For this purpose, conjugates SA2-BSA for SFZ and BSA–TN3C for TBZ were immobilized onto two different arrays at the following concentrations (one different per column of the array): 0, 1, 5, 10, 20 and 50 µg/mL. Each functionalized array was tested with three different concentrations of the corresponding monoclonal antibody: 0.5, 1 and 2 µg/mL. The combination that met the optimal trade-off was selected: for SFZ 10 µg/mL of SA2-BSA AE1 B28 conjugate with 2 µg/mL of 6C11 MAb and for TBZ 5 µg/mL of BSA–TN3C conjugate with 1 µg/mL of LIB–TN3C13 MAb. A comparison with the values obtained for the individual HFF-QCMD sensors has been included in Section S2 of the Supplementary Materials.

### 3.2. Standard Calibration Curves: Sample Pre-Treatment and Immunoassay Sensitivity

First, we studied the sample pre-treatment required by our HFF-QCMD array immunosensor to operate consistently. We started from the premise that a simple dilution of the honey sample in PBS could be adequate. We tested different dilutions (1/50, 1/100, 1/150 and 1/200 (*w*/*v*)) to evaluate the matrix effect of honey in the resonance frequency and dissipation measurements, as well as the occurrence of obstruction phenomena in the fluidic microchannels. For this purpose, a limiting MAb concentration for each analyte (see Section 3.1) was mixed with the honey dilutions. Then, each mixture was injected over the corresponding array region (see Figure 1c).

**Figure 3:**
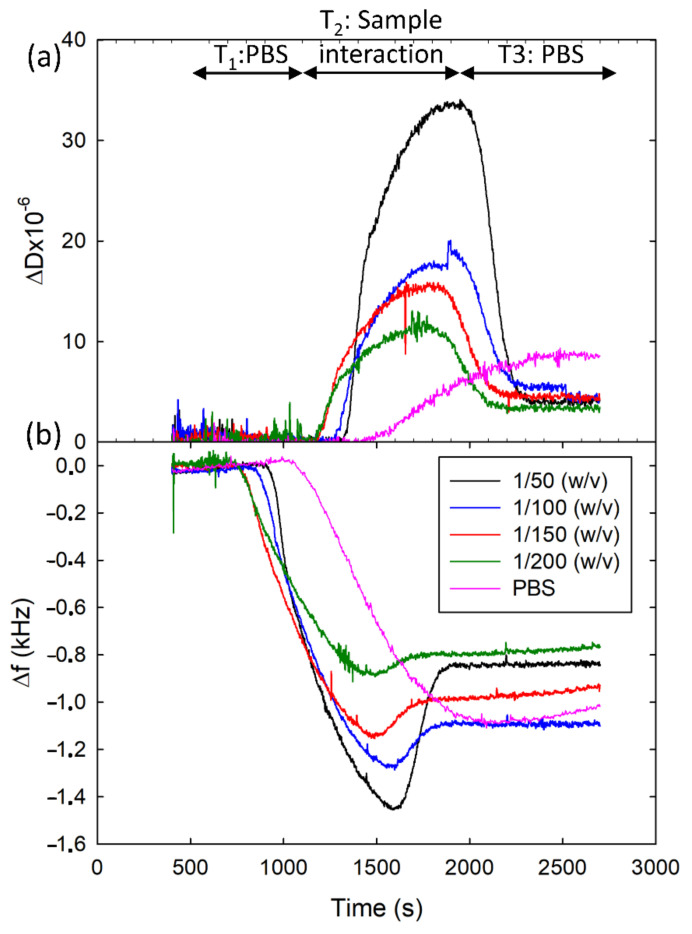
Records of Δ*D* (**a**) and Δ*f* (**b**) acquired to select the honey dilution ratio. Different curves correspond to PBS and different dilution ratios for SFZ analyte with 2 µg/mL concentration for the MAb without loss of generality.

Figure 3 shows the resonance frequency and dissipation shifts acquired at one of the HFF-QCMD sensors in the array for the different honey dilutions. The figure shows the results for SFZ analyte with 2 µg/mL concentration for MAb. Similar behavior was observed with TBZ. Before the sample injection, only PBS flowed through the array sensors (*T*_1_ in Figure 3) and a stable baseline was registered. During the interaction interval (*T*_2_ in Figure 3), the mixtures containing MAb and diluted honey came into contact with the array. Shifts in both resonance frequency and dissipation were observed and attributed to two different phenomena: (1) specific binding of the immunoreagents to the functionalized sensors surface and (2) honey viscoelasticity. As expected, the lower the dilution ratio the higher the sample viscoelasticity and hence the larger the frequency and dissipation shifts (minimum shifts in the interaction interval were registered for Mab + PBS mixture). Once honey dilution was completely replaced by PBS (*T*_3_ in Figure 3), only the effect of the immunoreagent interactions remained and, as expected in a gravimetric regime, dissipation barely changed. Only a small deviation ~4 × 10^−6^ persisted in Δ*D* around 25 min after the beginning of the interaction. This deviation tended to vanish with a PBS flow running over the time. Unlike dissipation response, a meaningful permanent decrease resulted in Δ*f* due to the antibody mass attached to the immobilized conjugate. In this case, all the records stabilized in a frequency ~1000 Hz ± 200 Hz (*T*_3_ interval). This 20% in variability remained even after regeneration, which led us to the conclusion that the differences were neither caused by the dilution ratio nor by the persistence of honey. We attribute these deviations to the small physical differences (i.e., Mesa region thickness and roughness) existing among the different resonators of the array [34], but also to the variability in the immobilization and regeneration processes applied over each sensor. Thus, no matrix effect affected our measurements in the stationary regime. However, we observed obstruction in the fluidic microchannels at lower dilution ratios, i.e., 1/50 (*w*/*v*). A 1/100 (*w*/*v*) ratio was found to be the optimum dilution, which is in the same order of magnitude as those used with individual HFF-QCMD resonators [31,32,33].

**Figure 4:**
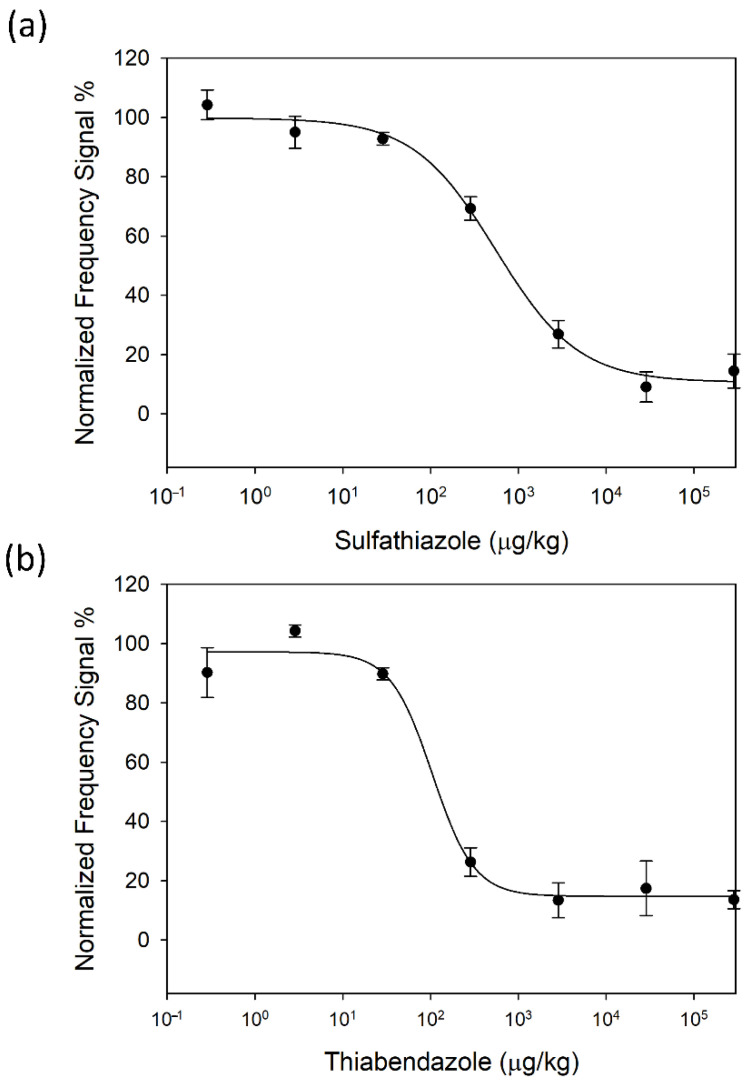
HFF-QCMD array standard calibration curves in honey diluted 1/100 (*w*/*v*) in PBS for SFZ (**a**) and TBZ (**b**). Each point is the average of eight determinations. Vertical bars represent standard deviations.

Once the sample pre-treatment protocol was defined, standard calibration curves were obtained for both SFZ and TBZ in 1/100 (*w*/*v*) diluted honey (see Figure 4a,b), respectively). Experimental dots and error bars in the figure correspond to the average of eight determinations of the same sample provided by eight sensors of the array, with their respective standard deviations. From these curves, the immunosensor analytical parameters of interest were determined (Table 1): Working Range (WR), LOD, LOQ and I_50_ value [33]. The limit of detection (LOD) corresponds to the analyte concentration that produces 10% inhibition of the maximum signal. The limit of quantification (LOQ) is obtained as the analyte concentration that produces 20% inhibition of the maximum signal. Finally, the working range is calculated as the range of concentrations that provide 20 and 80% of signal inhibition. These parameters are in the same order of magnitude as those obtained with individual HFF-QCMD technology [27,33]. We also obtained standard calibration curves with the array for both analytes in PBS. The results are in the same order of magnitude as those obtained with diluted honey, which reinforces our previous assumption that our measurements are not affected by any matrix effect due to honey.

LOD provided by the developed biosensor (31 µg/kg) reveals its capability for the detection of thiabendazole residues in honey down to the levels established by the current European legislation (MRL = 50 µg/kg). In the case of SFZ, since the legislation requires the absence of antibiotics, our technology can be used as a complementary screening technique to avoid sending those samples contaminated with concentrations above the LOD provided by our biosensor to centralized laboratories.

It is interesting to note that HFF-QCMD array technology requires similar immunoreagent volumes than those used with individual HFF-QCMD sensors. Furthermore, a 24-fold improvement in throughput together with the possibility of using the arrays around 15–20 times with reproducible behavior (see regeneration protocol described in Section 2.2.3) leads to a drastic reduction in costs.

### 3.3. HFF-QCMD Array Validation with HPLC LC-MS/MS

To evaluate the performance of HFF-QCMD array technology as an analytical tool, several samples of blank honey spiked at 4 levels (53, 105, 264 and 529 µg/kg) for SFZ and (31, 62, 156 and 313 µg/kg) for TBZ were analyzed by HFF-QCMD array and HPLC LC-MS/MS in terms of precision (Coefficient of Variation—CV%) and accuracy (recovery%).

**Figure 5:**
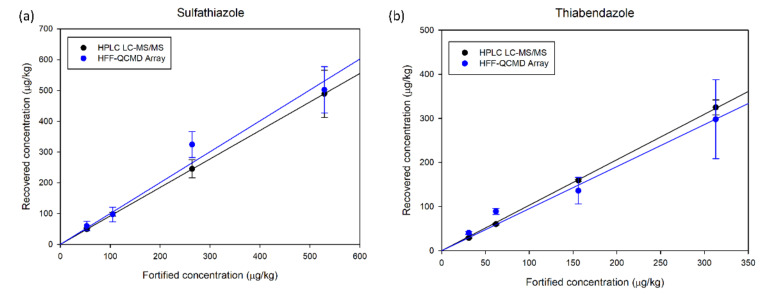
Recovered concentration vs. fortified known concentration for SFZ (**a**) and TBZ (**b**).

To compare the accuracy of the HFF-QCMD array technique with that of LC-MS/MS, their respective results of recovered concentration when applied to honey samples spiked with the mentioned TBZ and SFZ concentrations were correlated with the fortification levels (Figure 5a,b, respectively). Linear regressions performed provide correlation coefficients above 0.97 for both techniques and analytes. For TBZ, slopes of 0.905 and 1.044 were obtained for HFF-QCMD array and LC-MS/MS, respectively. Regarding SFZ, slopes of 0.973 and 0.924 for HFF-QCMD array and LC-MS/MS were obtained, respectively.

Table 2 and Table 3 show the comparison of the recovery and CVs obtained with both techniques for TBZ and SFZ, respectively. No false positives were detected in any case. Recovery percentages for HFF-QCMD array technique ranged from 87% to 143% and from 92% to 122% for TBZ and SFZ, respectively. Regarding CVs ranged from 7% to 30% for TBZ and from 13% to 25% for SFZ. In general, the precision and accuracy results for both analytes remained close to those established by the GC SANCO 12571/2013 guidelines [41], with an overall overestimation in the concentration values recovered for SFZ and an underestimation for TBZ. Our experimental results indicate that an effort is still needed in order to comply with the margins established by the standard, both for the recovery percentage (between 80% and 120%) and for CV (values lower than 20%).

These results reveal the capability of the HFF-QCMD array technique to provide simultaneous detection of analytes of a different chemical nature (i.e., pesticides and antibiotics) in the same honey sample, which is not possible with LC-MS/MS techniques. Furthermore, sample pre-treatment is very simple, thus, reducing analysis complexity and assay time. When compared with commercial systems currently used for honey screening, HFF-QCMD array technology presents important competitive advantages. Evidence Investigator™ Anti-Microbial Array II (Randox Laboratories Limited, Crumlin, County Antrim, UK) is based on a competitive chemiluminescent immunoassay and it has shown a good LOD (ranging from 1 to 10 ppb) for different antibiotics in Apulian honey samples [22]. Since SFZ has not been included in this study, a comparison with HFF-QCMD array sensitivity is not straightforward. However, it is possible to state that LODs provided by both technologies are in the range of tens of ppbs. As in the case of HFF-QCMD array technology, Evidence Investigator™ also allows for multiple analyte detection, but no pesticide results have been reported so far, and its assay protocol requires long incubation periods and repeated washing steps, which requires specialized personnel to be used. CHARM II (Charm Sciences, Inc., Lawrence, MA, USA), a multipurpose liquid scintillation counter (LSC), has also been used for honey screening [23]. CHARM II has achieved a 10 ppb LOD for sulfamethazine, a compound belonging to sulfonamides akin to SFZ. This sensitivity is slightly better than HFF-QCMD array device (31 ppb for SFZ), but it lies within the same order of magnitude. No results regarding pesticide detection have been reported using this system either. The CHARM II assay protocol is complex, especially for sulfonamides, where acid hydrolysis and reverse phase preparation are required to remove para-aminobenzoic acid interference and to release sulfathiazole sugar complexes [23]. Another important disadvantage of this system is that LSC technology requires the use of radioactive reagents that may be hazardous for the users and harmful to the environment.

## 4. Conclusions

In this work, we report the successful application of a novel 24 sensor HFF-QCMD biosensing array for the simultaneous detection (single assay) of TBZ and SFZ chemical residues in honey. The assay protocol, based on a competitive immunoassay, is completely automated so no trained personnel are required. LOD and LOQ are adequate for preliminary honey screening purposes. Unlike chromatographic methods, complex sample pre-treatment is not necessary, just a sample dilution (1/100 (*w*/*v*)) is required. This allows the simultaneous detection of compounds with very different chemical properties (i.e., pesticides and antibiotics), thus reducing the complexity, size and cost of the analysis.

All these characteristics hold promise for the fast adoption of this technology as an unattended on-line screening tool complementary to chromatographic analysis in the food packaging industry.

In the future, method optimization is envisaged by improving the microfluidic channel design to allow the injection of less diluted honey samples. A new HFF-QCMD array design will also be considered to work at higher resonance frequencies (200 MHz). Both strategies are expected to improve (decrease) LOD. Finally, higher accuracy and precision should be achieved by optimizing immobilization and regeneration processes.

## Figures and Tables

**Table 1:** Immunosensor analytical parameters for the HFF-QCMD array standard curves of SFZ and TBZ in 1/100 (*w*/*v*) diluted honey.

	SFZ (µg/kg) *	TBZ (µg/kg) *
$I_{50}$	554.6	104.6
LOD	53	31.3
LOQ	122.8	48.9
WR	122.8 to 2490.7	48.9 to 223.7

* µg/kg = ppb.

**Table 2:** Analysis of TBZ-spiked honey samples. Comparison of HFF-QCMD array technology with LC-MS/MS.

Spiked Level (μg/kg)	Recovered (μg/kg)	Recovery (%)	CV (%)	Detected (μg/kg)	Recovery (%)	CV (%)
	HFF-QCMD array ^1^			LC-MS/MS ^2^		
0	<LOD	No false positives		<LOD	No false positives	
31	40 ± 3	130	8	29 ± 1.2	94	4
62	89 ± 6	143	7	60 ± 1.6	97	2.6
156	135 ± 30	87	22	159 ± 2.4	102	1.5
313	297 ± 89	95	30	352 ± 17.2	104	5.3

Average of 8 and 6 independent determinations for HFF-QCMD array and LC-MS/MS, respectively. ^1^ Sample dilution factor 1/100 (*v*/*w*). ^2^ Sample dilution factor 1/2 (*v*/*w*).

**Table 3:** Analysis of SFZ-spiked honey samples. Comparison of HFF-QCMD array technology with LC-MS/MS.

Spiked Level (μg/kg)	Recovered (μg/kg)	Recovery (%)	CV (%)	Detected (μg/kg)	Recovery (%)	CV (%)
	HFF-QCMD array ^1^			LC-MS/MS ^2^		
0	<LOD	No false positives		<LOD	No false positives	
53	59 ± 14	112	25	49 ± 4	94	8.2
105	97 ± 23	92	24	98 ± 6.4	93	6.5
264	324 ± 42	122	13	245 ± 29.4	93	12
529	502 ± 75	94	14	489 ± 76.3	92	15.6

Average of 8 and 6 independent determinations for HFF-QCMD array and LC-MS/MS, respectively. ^1^ Sample dilution factor 1/100 (*v*/*w*). ^2^ Sample dilution factor 1/2 (*v*/*w*).

## Supplementary Materials

The following supporting information can be downloaded at: https://www.mdpi.com/article/10.3390/bios12060433/s1, Section S1. Chromatographic chemicals, immunoreagents and methodology, Section S2. Immunoassay optimization. Comparison with individual HFF-QCMD resonators.
