# Supplementary material for: High Fundamental Frequency (HFF) Monolithic Quartz Crystal Microbalance with Dissipation Array for the Simultaneous Detection of Pesticides and Antibiotics in Complex Food
Source: Biosensors (Basel). 2022 Jun 20;12(6):433. doi: 10.3390/bios12060433 (PMC9221314; doi:10.3390/bios12060433)
Supplement: Supplementary file 1 [file biosensors-12-00433-s001.zip › biosensors-1762916-supplementary.pdf]

biosensors

MDPI

Supplementary

High Fundamental Frequency (HFF) Monolithic Quartz Crystal Microbalance with Dissipation Array for the simultaneous detection of pesticides and antibiotics in complex food

María Calero[1,*], Román Fernández[1], María Garcia[2], Marisol Juan-Borrás[3], Isabel Escriche[3], Antonio Arnau[1], Ángel Montoya[1] and Yolanda Jiménez[1]

[1] Centro de Investigación e Innovación en Bioingeniería (Ci2B), Universitat Politècnica de València, Camino de Vera s/n, 46022 Valencia, Spain
[2] Advanced Wave Sensors S.L., Calle Algepser 24, 46988, Paterna, Valencia, Spain
[3] Instituto de Ingeniería de Alimentos para el Desarrollo (IIAD), Universitat Politècnica de València, Camino de Vera s/n, 46022, Valencia, Spain
* Correspondence: macaal3@teleco.upv.es

Citation: Calero, M.; Fernández, R.; García, M.; Juan-Borrás, M.; Escriche, I.; Arnau, A.; Montoya, Á.; Jiménez, Y. High Fundamental Frequency (HFF) Monolithic Quartz Crystal Microbalance with Dissipation Array for the Simultaneous Detection of Pesticides and Antibiotics in Complex Food. *Biosensors* **2022**, *12*, 433. https://doi.org/10.3390/bios12060433

Received: 24 May 2022
Accepted: 17 June 2022
Published: 20 June 2022

Publisher's Note: MDPI stays neutral with regard to jurisdictional claims in published maps and institutional affiliations.

Copyright: © 2022 by the authors. Licensee MDPI, Basel, Switzerland. This article is an open access article distributed under the terms and conditions of the Creative Commons Attribution (CC BY) license (https://creativecommons.org/licenses/by/4.0/).

S1. Chromatographic chemicals immunoreagents and methodology

HPLC-grade acetonitrile and methanol solvents were from Prolabo (VWR International, France). The formic acid (purity 99%), analytical grade sodium chloride (NaCl), anhydrous magnesium sulphate, disodium hydrogen citrate sesquihydrate, trisodium citrate dihydrate and Bondesil Primary-Secondary Amine (PSA) were provided by Sigma Aldrich (Saint Quentin Fallavier, France). Cartridges for SPME extraction (Strata X–CW, 100 mg/3 mL) were supplied by Phenomenex (California, USA).

Stock solutions of STZ and TBZ were prepared at 1000 μg mL^{-1} by dissolving 10 mg of each compound in 25 mL of methanol and stored at -20 ˚C. The working standard solutions were prepared by successive dilution of each stock solution with nanopure water. All of the standard solutions were stored in amber glass bottles at −18 °C for a maximum of one year.

The extraction and subsequent chromatography procedure for SFZ was carried out as described [1]. For TBZ analysis, the QuEChERS procedure was followed: a honey sample (5.0 g) was weighed into a polypropylene centrifuge tube (50 mL), and spiked when proceeded, with proper amounts of working standard solution of TBZ. Next, 10 mL nanopure water were added and shaken until a complete honey homogenization occured, then 10 mL of acetonitrile, 4 g magnesium sulfate anhydrous (MgSO4), 1 g sodium chloride (NaCl), 1 g sodium citrate dihydrate and 0.5 g di-sodium hydrogen citrate sesquihydrate were added and vigorously hand shaken for 1 min followed for 1 min in a vortex mixer. The tube was centrifuged at 4000 rpm for 5 min at 4 ºC. After centrifugation, in order to clean up the solution, 4 mL of the supernatant was transferred to another tube (10 mL) containing 1.5 g of magnesium sulfate and 150 mg primary secondary amine (PSA). In the same way as before, subsequent shaking and centrifugation steps were performed. Finally, 1000 µL of the upper solution was filtered (nylon 0.22 mm) prior to being analysed by high-performance liquid chromatography-tandem mass spectrometry (HPLC-MS/MS). The column used was an Atlantis T3 (2.1 x 100 mm, Waters). The mobile phase consisted in 0.1% formic acid in nanopure water as phase [A] and methanol as phase [B] with a flow rate of 0.3 mL/min. The gradient elution program was as follows: 0–1 min, 95%-50% A; 1–3 min, 95% A; 3–4.2 min, 30%–10% A; 4.2–4.3 min, 10%-90% A; 4.3–6 min, 90% A. The instrument conditions were as follows: the capillary voltage was set at 4000 V, whereas the drying gas temperature and sheath gas temperature were both 350 °C, with a drying gas flow of 12 L/min, the nebulizer pressure was set at 35 psi. The ions monitored by multiple reactions monitoring (MRM) were 321→194

and 321→152.

S2. Immunoassay optimization. Comparison with individual HFF-QCM resonators

Table S1. Optimal monoclonal antibody and conjugate assay concentrations for individual 100MHz HFF-QCMD sensors and for the 50MHz HFF-QCMD array.

**Analyte**	**Antibody concentration (µg/mL)**		**Conjugate concentration (µg/mL)**	
	Array	Individual	Array	Individual
Thiabendazole	1	1	5	20
Sulfathiazole	2	2	10	5

While optimal antibody concentration was the same for both analytes, conjugate concentrations differed from those of the individual. For TBZ, the concentration was four times smaller in the array than in the individual. In the case of SFZ, the concentration in the array doubled that use in the individual sensors.

References

1. Cervera-Chiner, L.; Jiménez, Y.; Montoya, Á.; Juan-Borrás, M.; Pascual, N.; Arnau, A.; Escriche, I. High Fundamental Frequency Quartz Crystal Microbalance (HFF-QCMD) Immunosensor for detection of sulfathiazole in honey. *Food Control* **2020**, *115*, 107296.
